# Supplementary material for: Aspartic Aminopeptidase Is a Novel Biomarker of Aggressive Chronic Lymphocytic Leukemia
Source: Cancers (Basel). 2020 Jul 12;12(7):1876. doi: 10.3390/cancers12071876 (PMC7408864; doi:10.3390/cancers12071876)
Supplement: Supplementary file 1 [file cancers-12-01876-s001.pdf]

Supplementary Materials

Aspartic Aminopeptidase is a Novel Biomarker of Aggressive Chronic Lymphocytic Leukemia

Pramath Kakodkar, Sanket More, Kinga Andras, Nikos Papakonstantinou, Sharon Kelly, Mohammad Adib Makrooni, Csaba Ortutay and Eva Szegezdi

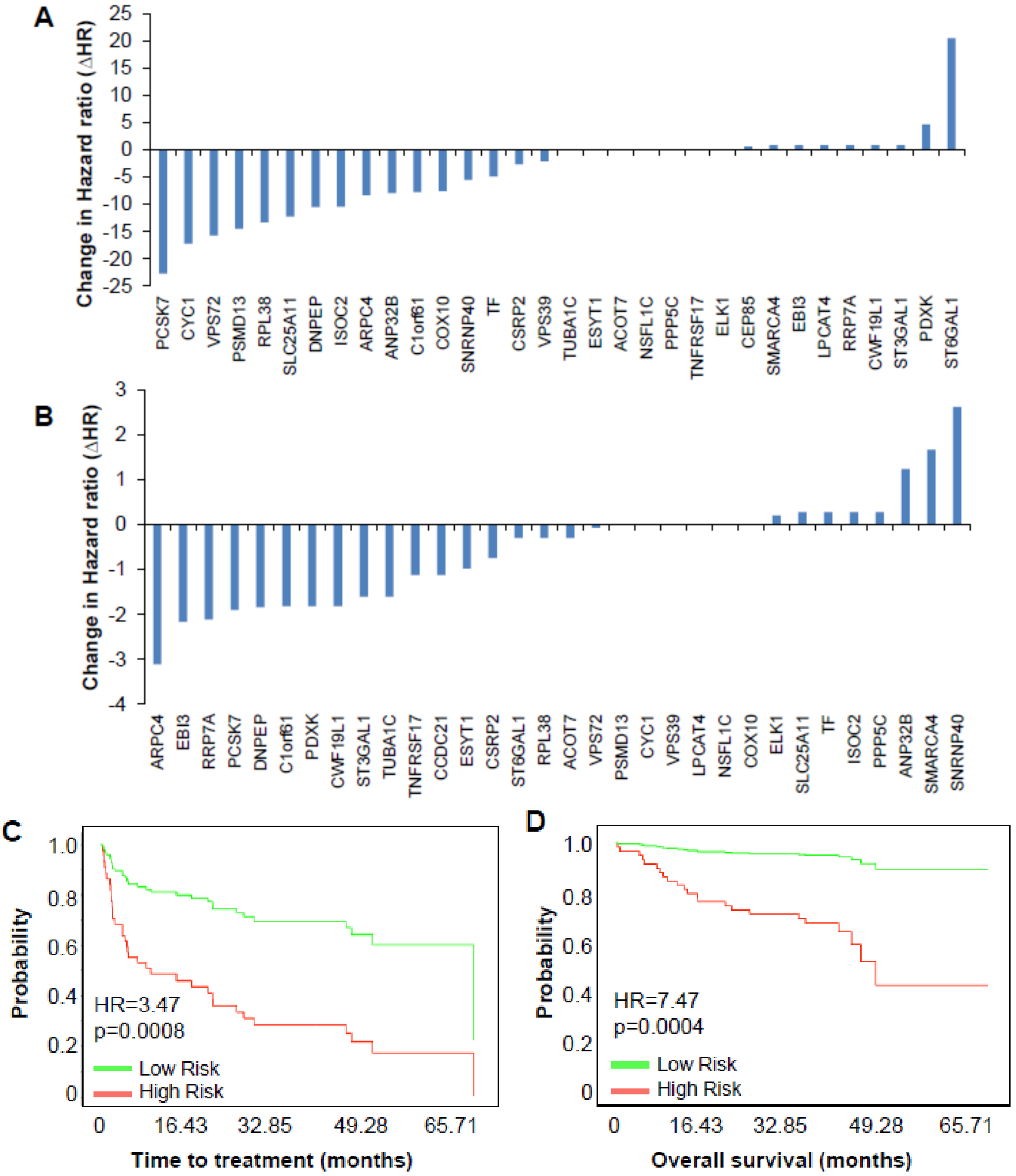

Figure S1. Effect of BCR-kinase correlating genes on time to treatment (**A**) and overall survival probability of CLL patients (**B**). The contribution of each of the 32 genes identified to correlate with the expression of BCR-signaling kinases was assessed by determining how they contribute to the hazard ratio (HR) of the 32 genes combined. The genes were iteratively removed from the 32-gene pool and the change in HR is plotted. (**C** and **D**) The combined prognostic potential of the top eight genes whose removal from the 32-gene set had the largest effect on the HR for time to treatment (**C**) and overall survival (**D**).

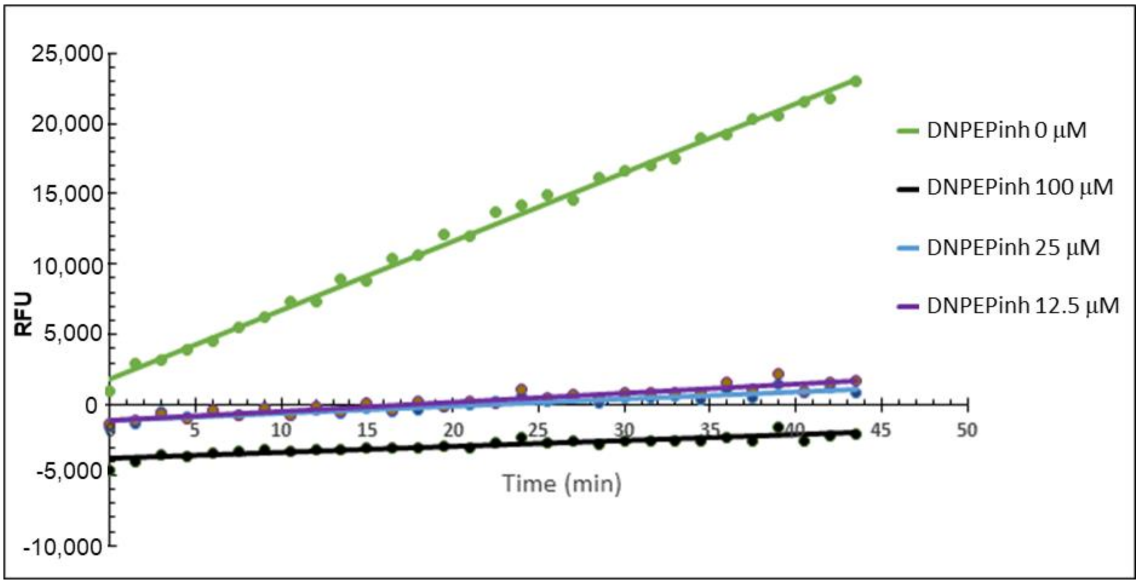

Figure S2. DNPEP inhibition by DI93293 enzyme kinetic assay. Mec-1 cell lysate was incubated with the indicated concentration of DI93293 for 30 minutes followed by addition of the DNPEP substrate, Asp-AMC. Cleavage of Asp-AMC was monitored over time by determining AMC fluorescence. Enzyme activity is graphed as relative fluorescence unit.

Table S1. Databases and selected samples for co-expression analysis.

GEO ID	Array ID	Platform Design	Number of Samples	Number of Genes	Experiment Description
GSE12626	E-GEOD-12626	Affymetrix GeneChip Human Genome U133A 2	150	12495	Transcriptomic analysis of irradiated immortalized B cells. Data were collected from lymphoblastoid cells at 0, 2 and 6 hours after exposure to 10 Gy of ionizing radiation on samples from 15 Centre d'Etude du Polymorphisme Humain pedigrees.
GSE20988	E-GEOD-20988	Agilent Whole Human Genome Microarray 4x44K 014850 G4112F	32 (two-channel)	19004	B cell lymphoma cell line (K1106) treated with JAK2 JMJD2C-shRNA or JAK2inhibitor. Trans-criptomic analysis at 24, 48, 72, 96 h (JAK2-shRNA), or 0.5, 2, 4, 6, 8, 16, 24, 48 h (JAK2-inh).
GSE21800	E-GEOD-21800	Affymetrix GeneChip Human Gene 1.0 ST Array	17	18868	Lymphoblastoid cell lines treated with different doses of hydrogen peroxide. Gene expression was measured at 0, 4, 12, 24 and 48 hours after treatment.
GSE22900	E-GEOD-22900	Agilent Whole Human Genome Microarray 4x44K 014850 G4112F	32 (two-channel)	19004	Gene expression in HBL-1 DLBCL cells treated with the IkB kinase beta inhibitor MLN120B measured at 2, 3, 4, 6, 8, 12, 16, and 24 h; and with JAK inhibitor I measured at 2, 4, 6 and 8 h.
GSE23591	E-GEOD-23591	Affymetrix GeneChip Human Gene 1.0 ST Array	40	18868	Study of primary B cell lymphoma and Hodgkin´s lymphoma cell lines. L1236 and MedB-1 lymphoma cells stimulated with IL13, gene expression measured at 0, 0.5, 1, 1.5, 2, 3, 4, 6, 8, 12 h.
GSE34176	E-GEOD-34176	Affymetrix GeneChip, HT Human Genome U133 HT_HG-U133A	29	8723	Transcriptomic analysis of two DLBCL cell lines DHL4 and DHL6 treated with SKY inhibitor, R406 or vehicle (DMSO) for 0, 2, 6, 24 or 48 hours.
GSE35163	E-GEOD-35163	Agilent Whole Human Genome Microarray 4x44K	64 (two-channel)	19004	Gene expression upon knockdown of the PI3K-linked transcription factor TCF3 or overexpression of its negative regulator ID3 in Burkitt's lymphoma cell lines (Namalwa, BL41, Daudi, Defauw, THOMAS). Gene expression monitored between 24 – 96 h after knockdown/ overexpression.
GSE36910	E-GEOD-36910	Affymetrix GeneChip Human Genome U133A 2.0	285	12494	Gene expression and co-expression network analysis in immortalised B cells isolated from 95 patients at baseline and after induction of cellular stress (ER stress, irradiation). Data were collected at 0, 2 and 6 hours after exposure to cellular stressors.
GSE39741	E-GEOD-39741	Agilent-026652 Whole Human Genome Microarray 4x44K v2	6 (two-channel)	15380	ABC-DLBCL cell line HBL1 treated with mepazine, a MALT1 inhibitor, that inhibits antiapoptotic NF-kB signalling. Gene expression was determined after 6, 12, and 24 h.
GSE41034	E-GEOD-41034	Agilent Whole Human Genome Microarray 4x44K	32 (two-channel)	19004	HBL1 and Oci-Ly3 DLBCL cells treated with the IkB kinase beta (IKKb) inhibitor MLN120B and with the MALT1 inhibitor zVRPR-fmk. Gene expression was monitored over time between 2-24 h.
GSE 43510	E-GEOD-43510	Affymetrix HT_HG-U133plust2	75	19345	Five DLBCL cell lines DHL4, DHL6, LY7, HBL1, U2932 treated with R406 SKY inhibitor. Gene expression was measured between 6 – 24 h.
GSE46971	E-GEOD-46971	Illumina HumanHT-12 V4.0 expression beadchip	16	21005	Gene expression measurements of the ABC DLBCL cell line HBL1 after NFKBIZ knockdown by shRNA for 24, 48, 72, 96 hours.
GSE46972	E-GEOD-46972	Illumina HumanHT-12 V4.0 expression beadchip	12	19042	Gene expression of HBL1 ABC DLBCL cell line after treatment with the IKKβ inhibitor MLN120b for 6, 12 and 24 h.
GSE46973	E-GEOD-46973	Agilent Whole Human Genome Microarray 4x44K 014850 G4112F	6 (two-channel)	19004	Gene expression in HBL-1 ABC DLBCL cell line after treatment with the MALT1 inhibitor, z-VRPR-fmk measured at 12, 24 or 48 h of treatment.

Table S2. List of genes correlating with ZAP-70 and at least two other BCR-signaling kinase genes.

Entrez ID	Gene symbol	Gene Name	Gene Inference						
			AKT1	AKT2	BTK	MAPK1	MAPK3	PI3KCD	ZAP-70
9159	PCSK7	Proprotein convertase subtilisin/ kexin type 7	▪	▪	▫	▪	▪	▪	▪
55968	NSFL1C	NSFL1 (p97) cofactor (p47)	▪	▫	▫	▪	▪	▪	▪
64793	CEP85	Centrosomal protein 85kDa	▪	▪	▫	▫	▪	▫	▪
1352	COX10	COX10 heme A: farnesyltransferase cyt. c oxidase assembly factor	▪	▫	▫	▫	▪	▪	▪
2002	ELK1	ETS Transcription Factor ELK1	▪	▫	▫	▫	▪	▪	▪
5536	PPP5C	Protein phosphatase 5, catalytic subunit	▪	▫	▫	▫	▪	▪	▪
11332	ACOT7	Acyl-CoA thioesterase 7	▫	▪	▫	▫	▪	▪	▪
79763	ISOC2	Isochorismatase domain containing 2	▫	▪	▫	▫	▪	▪	▪
5719	PSMD13	Proteasome (prosome, macropain) 26S subunit, non-ATPase, 13	▫	▫	▪	▪	▪	▫	▪
6597	SMARCA	SWI/SNF related, matrix associated, actin dependent regulator of chromatin, subfamily A, member 4	▪	▪	▫	▫	▪	▫	▪
6944	VPS72	vacuolar protein sorting 72 homolog	▪	▫	▫	▪	▫	▫	▪
84790	TUBA1C	tubulin, alpha 1c	▪	▫	▫	▫	▪	▫	▪
608	TNFRSF17	tumor necrosis factor receptor superfamily, member 17	▫	▪	▪	▫	▫	▫	▪
27341	RRP7A	ribosomal RNA processing 7 homolog A	▪	▫	▫	▫	▪	▫	▪
254531	LPCAT4	Lysophosphatidylcholine acyltransferase 4	▪	▫	▫	▫	▪	▫	▪
6169	RPL38	ribosomal protein L38	▫	▫	▪	▫	▪	▫	▪
55280	CWF19L1	CWF19 Like Cell Cycle Control Factor 1	▫	▫	▪	▫	▪	▫	▪
1466	CSRP2	cysteine and glycine-rich protein 2	▫	▫	▫	▪	▫	▪	▪
7018	TF	transferrin	▫	▫	▫	▪	▫	▪	▪
9410	SNRNP40	small nuclearribonucleo-protein 40kDa (U5)	▫	▫	▪	▪	▫	▫	▪
23339	VPS39	vacuolar protein sorting 39 homolog	▫	▪	▫	▫	▪	▫	▪
6482	ST3GAL1	ST3 beta-galactoside a-2,3-sialyltransferase 1	▫	▫	▫	▫	▪	▪	▪
10093	ARPC4	actin related protein 2/3 complex, subunit 4,20kDa	▫	▫	▫	▫	▪	▪	▪
1537	CYC1	cytochrome c-1	▫	▫	▫	▪	▪	▫	▪
8566	PDXK	pyridoxal (pyridoxine, vitamin B6) kinase	▫	▫	▫	▪	▪	▫	▪
6480	ST6GAL1	ST6 beta-galactosamide alpha-2,6-sialyltranferase 1	▫	▫	▫	▫	▪	▪	▪
8402	SLC25A11	Solute Carrier Family 25 Member 11	▫	▫	▫	▫	▪	▪	▪
10148	EBI3	Epstein-Barr virus induced 3	▫	▫	▫	▫	▪	▪	▪
10485	C1orf61	chromosome 1 open reading frame 61	▫	▫	▫	▫	▪	▪	▪
10541	ANP32B	acidic (leu-rich) nuclear phosphor-protein 32 family member B	▫	▫	▫	▫	▪	▪	▪
23549	DNPEP	aspartyl aminopeptidase	▫	▫	▫	▫	▪	▪	▪
23344	ESYT1	extended synaptotagmin like protein 1	▫	▫	▫	▫	▪	▪	▪

Table S3. Clinical data of patient samples used for qRT-PCRs.

**No. of Included Patients**	**34**
Median age, years (range)	64 (47–84)
Male sex, %	61.7
Evaluable for FISH, %	100
Del. 17p13, %	8.82
Evaluable for IGVH-status, %	100
IGVH mutated, %	79.12
IGVH unmutated, %	20.58
Median *IGVH* homology, %	95.4
Patients evaluable for Binet stage, %	100
Binet A at enrolment, %	82.3
Binet B at enrolment, %	8.82
Binet C at enrolment, %	8.82
Patients evaluable for Rai stage, %	100
Rai 0 at enrolment, %	58.82
Rai I at enrolment, %	23.52
Rai II at enrolment, %	5.88
Rai III at enrolment, %	8.82
Rai IV at enrolment, %	2.94
No. of newly diagnosed CLL, %	100
Treated at progression, %	41.17
Samples evaluable for OS, %	100
Deceased, %	29.41
CLL-unrelated death, %	11.76
Median follow-up, years (range)	5.48 (0–16)

Supplementary R-Script

Normalization of Ct values

```
my.target.genes<-
c("IGF2BP2","CLK1","NUP62","SLC39A14","ST3GAL1","ZAP70")
my.ref.gene<-"ABL1"
resfile<-'genewise_overexpression_with_efficiency.csv'
overexpr<-function(ct.target,ct.ref,eff.target,eff.ref){
rat <-eff.target^ct.target/eff.ref^ct.ref
return(rat)
}
eff <-data.frame(ABL1= 1.96,
IGF2BP2=1.91,
CLK1=1.99,
NUP62=1.93,
SLC39A14=1.99,
ST3GAL1=2.03,
ZAP70= 1.85)
reslist<-list()
for (t.genein my.target.genes){
datafile<-paste0(t.gene,'.csv')
my.dat <-read.csv(datafile)
my.dat[my.dat[,t.gene]==0,t.gene] <-NA
ct.means<-aggregate(my.dat[,2:3],
list(my.dat$Sample.ID), mean,na.rm=T)
names(ct.means)[1] <-'Sample.ID'
t.gene.oe<-paste0(t.gene,'.oe')
ct.means[,t.gene.oe]<-overexpr(ct.means[,t.gene],
ct.means[,my.ref.gene],
eff[,t.gene,],
eff[,my.ref.gene])
reslist[[t.gene]] <-ct.means[,c(1,4)]
}
98
library(plyr)
t.genes.oe<-join_all(reslist)
#t.genes.oe[complete.cases(t.genes.oe),]
write.csv(t.genes.oe,resfile)
```

© 2020 by the authors. Licensee MDPI, Basel, Switzerland. This article is an open access article distributed under the terms and conditions of the Creative Commons Attribution (CC BY) license (http://creativecommons.org/licenses/by/4.0/).
